# Crimean–Congo haemorrhagic fever in pregnancy: clinical outcomes and public health implications

**DOI:** 10.3389/fpubh.2025.1722564

**Published:** 2026-01-08

**Authors:** Edis Kahraman, Seyma S. Celina

**Affiliations:** 1 Department of Obstetrics and Gynecology, Acibadem Mehmet Ali Aydinlar University Atakent Hospital, Istanbul, Türkiye; 2 Center for Infectious Animal Diseases, Faculty of Tropical AgriSciences, Czech University of Life Sciences Prague, Prague, Czechia

**Keywords:** Crimean–Congo haemorrhagic fever, maternal-fetal health, one health, public health, zoonoses

## Abstract

Crimean–Congo haemorrhagic fever (CCHF) is the most widespread tick-borne viral disease of humans, with major public-health implications across western China, South Asia, the Middle East, south-eastern Europe, and Africa. Although uncommon, infection during pregnancy is often severe and associated with high maternal and fetal mortality. This mini review synthesizes 38 documented cases of CCHF in pregnancy to identify consistent patterns in clinical presentation, outcomes, and management. Maternal survival was recorded in 68% of cases, whereas fetal or neonatal survival was reported in only 39%. Ribavirin therapy has been associated with improved maternal outcomes in limited case reports, but its use in pregnancy remains restricted because of teratogenicity. Disease frequently mimicked obstetric emergencies such as HELLP syndrome, delaying diagnosis. CCHF during pregnancy presents substantial diagnostic and therapeutic challenges, including limited antiviral options and heightened risks of nosocomial transmission. Early recognition, timely virological confirmation, and strict infection-control measures are essential. Strengthening obstetric surveillance in endemic regions and developing pregnancy-safe therapeutics remain urgent priorities. Development of pregnancy-safe antivirals and integration of obstetric surveillance into endemic-area health systems are urgently needed.

## 1 Introduction

Crimean–Congo haemorrhagic fever (CCHF) is a severe viral disease caused by *Orthonairovirus* *haemorrhagiae*, commonly known as Crimean–Congo haemorrhagic fever virus (CCHFV), a member of the family *Nairoviridae*. Humans acquire infection primarily through bites of infected *Hyalomma* ticks or direct contact with the blood or tissues of viraemic animals or humans. With one of the broadest geographic ranges among arthropod-borne viruses, CCHFV is endemic across parts of Asia, the Middle East, south-eastern Europe, and Africa (1).

CCHFV infection is characterised by a sudden onset of high fever, myalgia, gastrointestinal disturbance, and haemorrhagic manifestations (2). In severe cases, uncontrolled bleeding may lead to death, usually between days 7 and 9 of illness. Because of its high transmissibility, absence of licensed antiviral treatment, and potential for nosocomial spread, CCHFV is classified as a biosafety level-4 (BSL-4) pathogen (2).

Pregnancy introduces a distinct clinical challenge. Gestation-associated immune tolerance increased vascular permeability, and a procoagulant yet consumptive haemostatic state may amplify viral endothelial injury and haemorrhagic complications, exacerbating disease severity (3). Clinical overlap with conditions such as haemolysis, elevated liver enzymes, and low platelets (HELLP) syndrome often leads to delayed or missed diagnosis (4).

**Figure 1:**
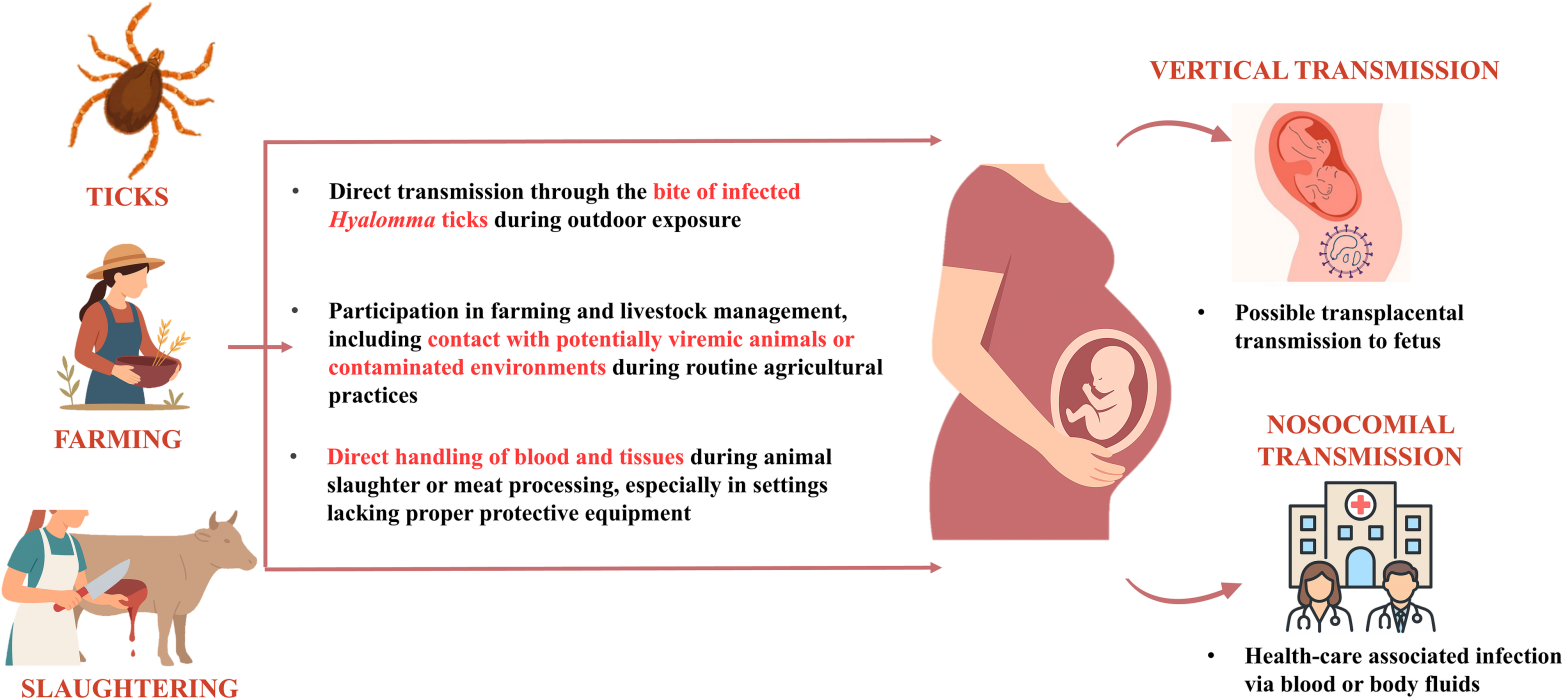
Transmission pathways of Crimean-Congo haemorrhagic fever virus (CCHFV) infection in pregnancy. Pregnant women may acquire CCHFV through tick bites (particularly from *Hyalomma* species), direct exposure during agricultural labor involving livestock, or through the handling and slaughtering of infected animals. Once infected, vertical transmission to the fetus is theoretically possible via transplacental spread. In addition, maternal infection poses a risk of nosocomial transmission to healthcare workers, especially during labor and delivery, through contact with infected blood or body fluids. This schematic is based on transmission mechanisms described in the published literature (1, 18).

Pregnant women may acquire infection through various exposures, including *Hyalomma* tick bites during outdoor activity, agricultural or livestock work involving contact with potentially viraemic animals, or handling of animal tissues and blood during slaughter without protective measures (Figure 1). Vertical transmission is biologically plausible and has been reported, although its frequency remains uncertain. Maternal infection also poses risks for nosocomial spread during delivery or invasive procedures (5).

Arthropod-borne viruses, including CCHFV, increasingly threaten maternal and fetal health worldwide as climate change, vector expansion, and urban–rural interface dynamics alter exposure patterns (6). CCHFV infection during pregnancy is associated with disproportionately high maternal and fetal mortality, and may mimic obstetric emergencies (5, 7). Severe maternal disease can compromise placental perfusion and oxygenation, while direct placental infection and intrapartum exposure contribute to poor neonatal outcomes (6).

Therapeutic options remain limited. Ribavirin, the most widely used antiviral against CCHFV, is teratogenic and generally contraindicated in pregnancy except in life-threatening cases (8). Passive immunisation with CCHFV-specific hyperimmune globulin has shown promise in experimental models, but data in pregnant patients are lacking (9, 10).

The expanding geographical distribution of *Hyalomma* ticks, climate-driven ecological shifts, and increased rural–urban interface dynamics raise concern for greater exposure among pregnant women in endemic regions. This review consolidates available evidence on CCHF in pregnancy, summarizes maternal and fetal outcomes, and highlights priorities for clinical management and public-health preparedness.

## 2 Methods

A literature search was conducted using the PubMed database on 28 February 2025 with the following Boolean query: [(Crimean Congo Haemorrhagic fever) OR (Crimean Congo Haemorrhagic fever) OR (CCHF) AND (pregnancy)]. Only English-language publications were included, and duplicate records were removed. A total of 15 relevant publications were identified, encompassing 38 documented cases of CCHF in pregnancy. Extracted variables included maternal and fetal outcomes, gestational age at the time of infection, and the administration of antiviral therapy, particularly ribavirin. The data were systematically analyzed to identify patterns and potential clinical implications relevant to the management of CCHF in pregnancy.

Only laboratory-confirmed cases of CCHF were included in this review, defined as diagnosis by PCR or serology (IgM positivity and/or rising IgG titers). In early historical reports or fatal cases where PCR was unavailable, confirmation by viral culture or immunohistochemistry was accepted.

## 3 Epidemiology and clinical presentation

**Figure 2:**
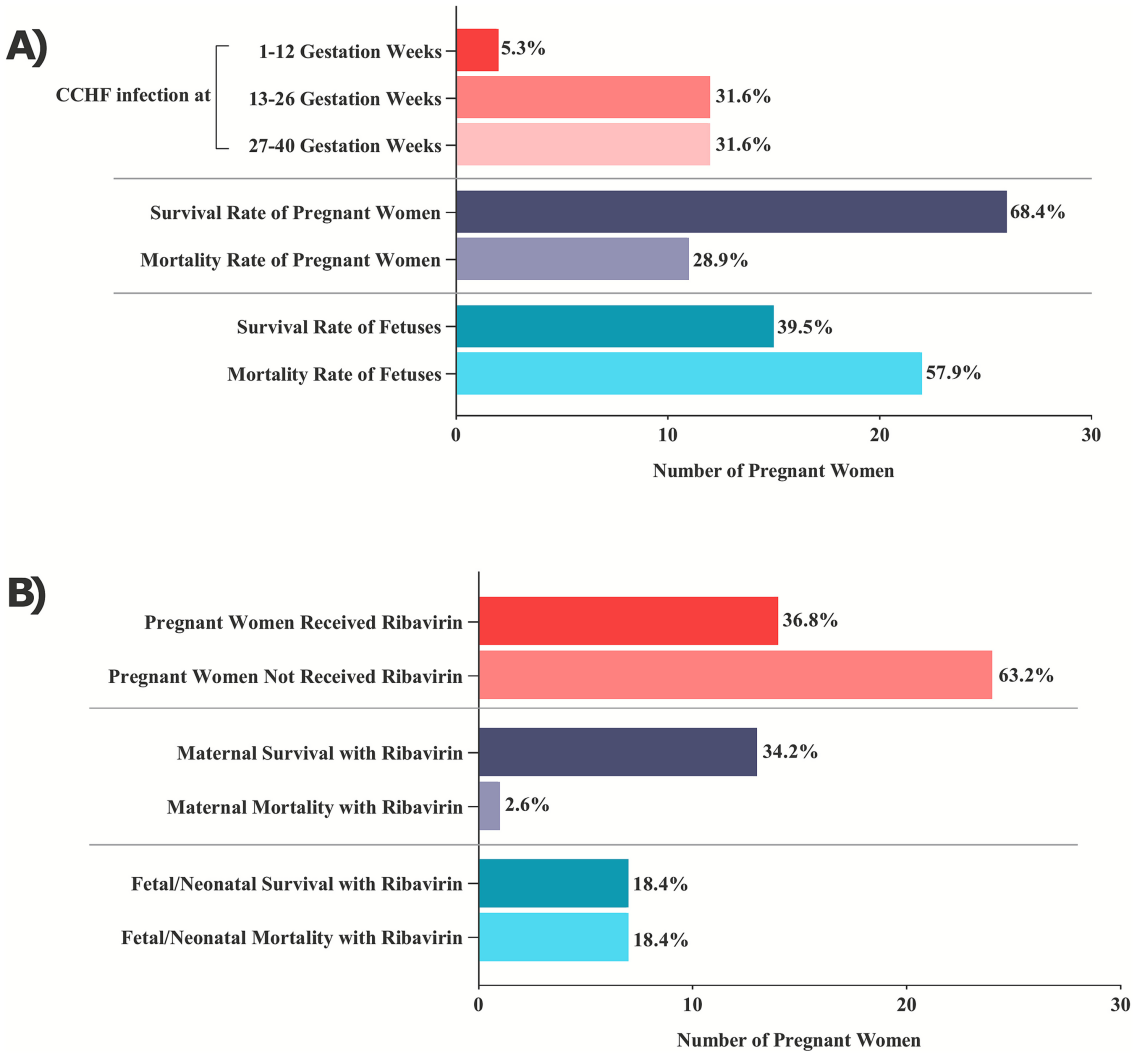
Quantitative summary of published pregnancy-associated CCHF cases. The figure is based on aggregated case data from published reports and is intended for conceptual visualization rather than representation of raw or individual-level data. **(A)** Heterogeneity in CCHF cases along gestational periods and maternal-fetal/neonatal outcomes. **(B)** Impact of ribavirin therapy on both maternal and fetal health during CCHF infections in pregnancy.

Available case reports show that the clinical course of CCHF during pregnancy is highly variable, with outcomes ranging from full maternal recovery to fatal haemorrhage. Across 38 documented cases, infection occurred in all trimesters, indicating that CCHF can develop at any stage of gestation (Table 1; Figure 2).

**Table 1:** Characteristics of 38 reported cases of CCHF infection during pregnancy in literature.

Author, Year of publication	Country, Year(s)	Number of pregnant women with CCHFV	Age	Gestational age	Diagnostic method used	Maternal outcome		Fetal/neonatal outcome	Ribavirin treatment
Al-Tikriti et al. 1981	Iraq, 1979	3	ND	ND	Viral culture/Clinical	Died	66.6% mortality	Died	No
						Died		Died	
						Survived		Died	
Nabeth et al. 2004	Mauritania, 2003	2	30	ND	PCR/Serology	Died	50% mortality	Died	No
			ND	ND	PCR/Serology	ND		ND	No
Sharif-Mood et al. 2007	Iran, 2000–2005	6	19–38	ND	PCR/Serology	Survived	16.7% mortality	Died	Yes
				ND	PCR/Serology	Survived		Died	Yes
				ND	PCR/Serology	Survived		Died	Yes
				ND	PCR/Serology	Survived		Survived	Yes
				ND	PCR/Serology	Survived		Survived	Yes
				16	PCR/Serology	Died		Died	Yes
Dizbay et al. 2009	Turkey, 2009	1	22	36	PCR/Serology	Survived		Survived	Yes
Aydemir et al. 2010	Turkey, 2009	1	29	30	PCR	Survived		Survived	No
Ergonul et al. 2010	Turkey, 2003–2008	3	40	38	Serology	Survived	33.3% mortality	Died	Yes
			20	19	PCR	Survived		Died	No
			28	28	PCR/Serology	Died		Died	No
Naderi et al. 2010	Iran, 2009	2	ND	ND	Clinical	Died	100% mortality	Died	No
			31	ND	Clinical	Died		Died	No
Mardani et al. 2013	Iran, 2011	1	24	16	PCR/Serology	Survived		Survived	Yes
Unlusoy-Aksu et al. 2014	Turkey, 2014	1	23	36	PCR	Survived		Survived	Yes
Gozel et al. 2014	Turkey, 2007–2011	5	35	8	PCR/Serology	Survived		Died	No
			30	18	PCR/Serology	Survived		Survived	No
			41	20	Serology	Survived		Survived	No
			19	21	PCR/Serology	Survived		Survived	No
			27	32	PCR/Serology	Survived		Survived	No
Duygu et al. 2015	Turkey, 2011	2	25	17	PCR	Survived		Survived	No
			22	20	PCR	Survived		Survived	No
Pschenichnaya et al. 2015	Russia, 2011	1	23	22	PCR/Serology	Died	100% mortality	Died	No
Pschenichnaya et al. 2017	Russia, 2002	8	20	16	PCR/Serology	Survived	37.5% mortality	Survived	No
	Russia, 2003		19	38	Serology	Survived		Survived	Yes
	Russia, 2004		17	30	PCR	Died		Died	No
	Russia, 2005		24	17	Serology	Survived		Died	No
	Kazakhstan, 2009		23	40	Immunohistochemistry	Died		Died	No
	Kazakhstan, 2010		21	34	Immunohistochemistry	Died		Died	No
	Russia, 2011		17	18	PCR	Survived		Survived	Yes
	Turkey, 2016		20	4	PCR	Survived		Died	No
Dedkov et al. 2017	Russia, 2016	1	26	34	PCR	Survived		Died	Yes
Ajazaj-Berisha et al. 2025	Kosovo, 2013	1	29	36	PCR	Survived		Died	Yes

Early-trimester infections were rare, with only two cases (5.3%) reported at 4 and 8 weeks of gestation (5, 7). Second-trimester infections were more common, representing 12 cases (31.6%) and clustering between 16 and 22 weeks (3, 5, 7, 11–14). Third-trimester infections accounted for another 12 cases (31.6%), often presenting with severe or fulminant disease (3–5, 7, 8, 15–17). In 12 cases (31.6%), gestational age was not reported.

Across all 38 cases, maternal survival was 68.4%, whereas fetal or neonatal survival was only 39.5%, indicating substantial fetal vulnerability even when mothers survived. Ribavirin was administered in 14 pregnancies (36.8%), and maternal survival was recorded in 13 of these (34.2% of all cases; 92.9% of ribavirin recipients). A recent late-pregnancy case from Kosovo illustrates the diagnostic difficulty in this setting: initial misclassification as HELLP syndrome delayed CCHF recognition, and although the mother survived with intensive care, the neonate died on the sixth postpartum day (4).

Of the 38 cases, gestational age was reported in 26 pregnancies. Among these, maternal survival was highest in the first trimester (100%) and declined through the second (83.3%) and third trimesters (66.7%). Fetal or neonatal survival showed a similar pattern, with 0% survival in first-trimester infections, 66.7% survival in the second trimester, and 41.7% survival in the third trimester. These findings suggest that advancing gestational age may increase both maternal physiological vulnerability and fetal risk, likely due to greater haemodynamic strain, more extensive placental involvement, and a higher probability of intrapartum exposure when maternal illness begins late in pregnancy.

## 4 Maternal–fetal outcomes and therapeutic considerations

CCHF during pregnancy reflects the interaction between viral pathogenicity and the physiological changes of gestation (6). Pregnancy-related immune modulation, vascular alterations, and haemostatic shifts can intensify disease severity. The shift toward a Th2-dominant immune profile reduces antiviral cytotoxic responses and may permit greater viral replication and prolonged viraemia (6, 18). Increased vascular permeability and endothelial activation can further enhance CCHFV-mediated endothelial injury, which may lead to more severe plasma leakage, haemorrhage, and multiorgan dysfunction (18). Hepatic involvement, driven by viral infection of hepatocytes and the increased metabolic demands of pregnancy, contributes to elevated transaminases and coagulopathy (2, 19).

These immune, vascular, and hepatic disturbances can mimic obstetric complications such as HELLP syndrome or severe preeclampsia, conditions that also involve microangiopathic haemolysis, endothelial dysfunction, and hepatic injury. This overlap contributes to frequent misdiagnosis and highlights the importance of timely virological testing to distinguish CCHF from other life-threatening maternal disorders (3, 4, 20).

Case reports consistently link CCHFV infection with high risks of maternal death, fetal loss, and serious obstetric complications. Prognosis appears to vary by gestational age, with first-trimester infections often ending in miscarriage, whereas late-pregnancy infections more commonly result in neonatal death shortly after birth (3, 5, 7, 8, 15–17). Most cases involved environmental or occupational exposures such as farming, livestock handling, or participation in slaughtering activities, highlighting the need for preventive education in endemic areas (3, 5, 7, 8, 11, 15, 16, 20, 21).

Ribavirin, a guanosine analogue used for respiratory syncytial virus and hepatitis C infections, has been administered for CCHF for more than two decades (22). Although it may improve maternal outcomes, its teratogenic potential limits use to life-threatening circumstances in which maternal benefit outweighs fetal risk. Supportive care, which includes management of haemorrhage, thrombocytopenia, hepatic dysfunction, and multiorgan failure, remains the primary therapeutic approach (23).

The infection also poses a significant public-health concern. Nosocomial transmission has occurred during obstetric procedures and invasive clinical care (5, 13, 20, 21). These events emphasize the need for strict infection-prevention measures in maternity settings and consistent use of personal protective equipment. Early recognition, multidisciplinary management, and improved health-system preparedness are crucial to achieving better maternal and neonatal outcomes in endemic regions.

## 5 Country-specific epidemiological patterns and maternal–fetal implications

Regional variation in reported CCHF cases reflects differences in diagnostic readiness, health-system capacity, and clinician awareness. Across endemic regions, case reports consistently demonstrate high maternal and fetal mortality, uncertain therapeutic benefit, and notable risk of nosocomial transmission, particularly where diagnosis is delayed or infection-control measures are insufficient. Improved outcomes are closely linked to timely PCR-based diagnosis and access to intensive supportive care (5, 21). Regions with rapid recognition, most prominently Turkish endemic provinces, were more likely to initiate early haemorrhage control, transfusion support, and coordinated multidisciplinary management, contributing to higher survival (3, 5, 7). In contrast, cases from cases from Iran, Iraq, Kazakhstan, and Russia often involved delayed or incorrect initial assessment due to failure to recognize CCHF early, leading to late ICU admission and inadequate supportive care, and these delays were consistently associated with poorer maternal and fetal outcomes (5, 20). These patterns show that prognosis depends not only on viral factors but also on clinician awareness, diagnostic capacity, and access to well-resourced critical care.

Geographic comparison highlights substantial variation across affected regions. Early cases from Iraq (1979) showed extremely high maternal mortality (66.6%) and fetal loss, likely reflecting the absence of diagnostic and treatment protocols at the time (24). Mauritania (21) reported similarly severe outcomes, including a maternal death and nosocomial transmission after animal exposure, indicating the vulnerability of low-resource rural settings (21). In Iran, six cases from 2000 to 2005, largely associated with meat handling, resulted in four fetal losses and one maternal death despite ribavirin use (14). Additional cases in 2009 and 2011 included maternal fatalities and secondary infections among healthcare workers, although early ribavirin therapy in one 16-week pregnancy resulted in survival of both mother and fetus (12, 20). Overall, Iran demonstrated an intermediate severity profile, with approximately one-third maternal mortality and frequent fetal loss, often linked to delayed recognition or misinterpretation of symptoms as obstetric complications.

Turkey has reported the largest number of cases, with outcomes influenced by gestational age and access to timely care. Early reports described a woman at 38 weeks who survived while her neonate died, a 19-week case ending in miscarriage despite maternal recovery, and a 28-week case in which both mother and fetus died (3). Ribavirin use was inconsistent: treatment at 36 weeks led to survival of both mother and infant—the first such outcome in Turkey—whereas several untreated mid-gestation cases had mixed fetal results (8). Subsequent series noted maternal survival in all five untreated cases but fetal survival in only three (7). Two additional livestock-associated cases in 2011 showed survival of both mother and infant (11). Later reports documented favourable maternal and fetal outcomes with ribavirin in late gestation (17), contrasted by a 2016 first-trimester infection resulting in fetal loss despite maternal survival (5). Overall, Turkey demonstrated comparatively better maternal (81%) and fetal (56%) survival, likely reflecting enhanced clinician awareness, earlier PCR-based diagnosis, and more consistent access to supportive care.

In Russia, isolated and outbreak-related cases have been documented. A 2011 infection at 22 weeks resulted in maternal and fetal death and transmission to eight healthcare workers, raising concern about aerosol-generating procedures during clinical care (13). Earlier cases (2002–2005) suggested improved outcomes with ribavirin (5), but later reports were variable, including complete maternal–fetal survival at 18 weeks and fetal death at 34 weeks despite maternal recovery (16). In Kazakhstan, severe infections in 2009 and 2010 resulted in maternal and neonatal deaths, followed by nosocomial transmission among caregivers (5). The most recent case—36 weeks in Kosovo (2013), published in 2025—was initially misdiagnosed as HELLP syndrome; despite successful maternal recovery following intensive supportive care, the neonate died on the sixth postpartum day (4).

Across all regions, recurring patterns include high maternal and fetal mortality, outcome differences linked to gestational timing, uncertain but occasionally favourable effects of ribavirin therapy, and frequent nosocomial transmission in obstetric settings. Effective management therefore relies on rapid diagnosis, individualized clinical strategies, and strict infection-prevention protocols. Taken together, regional disparities indicate that outcomes depend not only on viral virulence but also on diagnostic familiarity, health-system preparedness, and timely access to intensive supportive care.

## 6 Breastfeeding and postnatal transmission risk

The potential for CCHF transmission through breastfeeding remains a critical but understudied aspect of maternal and neonatal care. Although nosocomial and horizontal transmission of CCHFV has been well documented (5, 13, 20, 21, 25), evidence for viral shedding in breast milk is extremely limited.

A report from Turkey provides rare insight into this question, describing two lactating women diagnosed with CCHF who continued breastfeeding until hospital admission (26). The first, an 18-year-old with a history of livestock exposure and unprotected tick removal, tested positive for CCHFV RNA in blood but negative in breast milk; her 3-month-old infant, breastfed until admission, remained asymptomatic with normal laboratory findings. The second, a 24-year-old woman with a recent tick bite, also had CCHFV RNA detected in serum but not in breast milk; her 5-month-old infant showed no evidence of infection. These findings suggest a low likelihood of viral transmission through breast milk, although theoretical mechanisms such as microscopic nipple trauma or subclinical mastitis could permit viral passage (26).

Current clinical guidance does not list CCHF as a contraindication to breastfeeding, reflecting the limited and inconclusive nature of available data. Nevertheless, given the high severity of maternal infection and potential neonatal risks, temporary suspension of breastfeeding during the maternal viraemic phase may be prudent until further evidence clarifies safety (27). Further investigation is urgently required to define evidence-based recommendations that balance infection prevention with the nutritional and psychological benefits of breastfeeding.

## 7 Public-health implications and prevention

The infection poses both an urgent clinical concern for affected mothers and a broader public-health challenge in endemic regions (28). High case-fatality rates, the possibility of vertical and nosocomial transmission, and the lack of pregnancy-safe antiviral options highlight the need for coordinated prevention strategies across clinical, veterinary, and environmental health sectors.

Women of reproductive age in rural or pastoral settings face elevated risk due to agricultural labour, livestock handling, and involvement in slaughtering or butchering activities (29). Education on tick avoidance, safe livestock practices, and early medical evaluation after tick bites can significantly reduce exposure (18). Integrating these preventive messages into routine antenatal care programmes in endemic areas may provide a practical and cost-effective approach.

Health-system readiness is equally important. Outbreaks affecting obstetric units show the importance of early case recognition, reliable laboratory diagnostics, and strict infection-prevention protocols. Essential measures include barrier nursing and appropriate personal protective equipment during delivery and other invasive procedures. Including the infection in the differential diagnosis of febrile pregnant women with thrombocytopenia or bleeding symptoms is critical to prevent misclassification as HELLP syndrome or other obstetric disorders.

At the surveillance level, stronger collaboration between human and veterinary sectors can improve detection of viral activity in livestock and ticks, supporting better risk forecasting and targeted preventive messaging (30). Developing safe antiviral therapies and vaccines suitable for use during pregnancy remains an important research priority. Until such tools are available, improved infection-control capacity, enhanced community awareness, and integrated One Health surveillance remain the most effective strategies to reduce maternal and neonatal mortality linked to this high-risk pathogen.
